# Impaired Spatial Learning Strategies and Novel Object Recognition in Mice Haploinsufficient for the Dual Specificity Tyrosine-Regulated Kinase-1A (Dyrk1A)

**DOI:** 10.1371/journal.pone.0002575

**Published:** 2008-07-02

**Authors:** Glòria Arqué, Vassiliki Fotaki, David Fernández, María Martínez de Lagrán, Maria L. Arbonés, Mara Dierssen

**Affiliations:** 1 Genes and Disease Program, Genomic Regulation Center-CRG, Pompeu Fabra University, Barcelona, Spain; 2 CIBER de Enfermedades Raras (CIBERER), Barcelona, Spain; Centre National de la Recherche Scientifique, France

## Abstract

**Background:**

Pathogenic aneuploidies involve the concept of dosage-sensitive genes leading to over- and underexpression phenotypes. Monosomy 21 in human leads to mental retardation and skeletal, immune and respiratory function disturbances. Most of the human condition corresponds to partial monosomies suggesting that critical haploinsufficient genes may be responsible for the phenotypes. The *DYRK1A* gene is localized on the human chromosome 21q22.2 region, and has been proposed to participate in monosomy 21 phenotypes. It encodes a dual-specificity kinase involved in neuronal development and in adult brain physiology, but its possible role as critical haploinsufficient gene in cognitive function has not been explored.

**Methodology/Principal Findings:**

We used mice heterozygous for a *Dyrk1A* targeted mutation (Dyrk1A+/−) to investigate the implication of this gene in the cognitive phenotypes of monosomy 21. Performance of Dyrk1A+/− mice was assayed 1/ in a navigational task using the standard hippocampally related version of the Morris water maze, 2/ in a swimming test designed to reveal potential kinesthetic and stress-related behavioral differences between control and heterozygous mice under two levels of aversiveness (25°C and 17°C) and 3/ in a long-term novel object recognition task, sensitive to hippocampal damage. Dyrk1A+/− mice showed impairment in the development of spatial learning strategies in a hippocampally-dependent memory task, they were impaired in their novel object recognition ability and were more sensitive to aversive conditions in the swimming test than euploid control animals.

**Conclusions/Significance:**

The present results are clear examples where removal of a single gene has a profound effect on phenotype and indicate that haploinsufficiency of *DYRK1A* might contribute to an impairment of cognitive functions and stress coping behavior in human monosomy 21.

## Introduction

Aneuploidies are associated with several human diseases that affect specific brain areas, leading to mild or severe mental retardation. However, extensive research is needed to establish how the loss and/or gain of genetic material might contribute to the development of these disorders [1]. Aneuploidy diseases resulting from the loss of specific chromosomal segments have been assumed to arise either from single critical haploinsufficient genes [2] or the accumulated effects of many subcritical genes [3]. Monosomy 21 is a rare form of aneuploidy associated with several phenotypes, including mental retardation, intrauterine and postnatal growth retardation, hypertonia, facial dysmorphism, cardiac anomalies, and microcephaly [4], [5]. Most of the detected cases of monosomy 21 correspond to partial monosomies, suggesting that critical chromosomal regions or haploinsufficient genes may be responsible for the observed phenotypes. However, to date, relatively few of those genes have been identified that can be linked to specific phenotypes, and specifically to mental retardation. The clinical phenotype of patients varies according to the size and location of the deleted chromosomal fragment [6]. Ehling et al. [7] reported two unrelated patients with partial monosomy of 21q22.2-q22.3 who presented minor dysmorphic features and mild mental retardation. A more detailed study of unrelated individuals with overlapping partial deletions of chromosome 21 [4], [8] has indicated an 8.4-Mb region in chromosome band 21q22.2–22.3 (KCNJ6-COL6A2) as responsible for cortical dysplasia and mental retardation leading to the proposal that one or more dosage-sensitive genes in this region contribute to cortical development and cognition.

The *DYRK1A* gene is localized within the human chromosome 21q22.2 region [9], [10]. It is the mammalian homologue of the *Drosophila minibrain* (*mnb*) gene that is essential for normal postembryonic neurogenesis [11]. The human (*DYRK1A*) and rodent (*Dyrk1A*) genes are ubiquitously expressed in fetal and adult tissues, with strong expression in brain [12]–[17]. Recently, two unrelated patients have been identified with prenatal onset of microcephaly, intrauterine growth retardation, feeding problems, developmental delay, and febrile seizures/epilepsy who both carry a *de novo* balanced translocation that truncates the *DYRK1A* gene at chromosome 21q22.2 [18]. Of interest for the mental retardation phenotype, *Dyrk1A* shows high levels of protein expression in the limbic system [15], including the hippocampus, a structure that plays a critical role in the processes of emotional behavior, motivation, and learning and memory.

Previous studies in model organisms suggest that *Dyrk1A* may be a critical dosage-sensitive gene involved in behavioral and cognitive phenotypes [19]–[21]. *Drosophila* flies that carry mutations in the *mnb* gene express 30–60% of wild type mnb protein levels and display a specific and marked size reduction in specific brain areas with no gross alterations in neuronal architecture and a behavioral phenotype showing reduced locomotor activity and poor odor discrimination [11]. In mice, haploinsufficiency for *Dyrk1A* leads to decreased neonatal viability and reduced body size from birth to adulthood [19]. Neurobehavioral analysis revealed preweaning developmental delay of heterozygous Dyrk1A+/− mice and specific motor deficits in adults [20]. In addition, brains of these mice are decreased in size in a region-specific manner, and the microarchitecture of pyramidal cells in the layer III of the cerebral cortex is markedly altered, with reduction of dendritic arborization and of spine density [22]. However, the involvement of *Dyrk1A* in cognition has only been investigated in transgenic mouse models, overexpressing the gene. These studies showed a clear alteration of the learning and memory phenotypes [21], [23]–[25]. Similar phenotypes have been also detected in trisomy Down syndrome mouse models bearing *Dyrk1A* [26]–[28].

The chromosomal location of human *DYRK1A*, along with the phenotypic defects observed in the Dyrk1A+/− mutant mice that carry one copy of the murine homologue, suggest that *DYRK1A* might be a good candidate gene for some of the cases of partial monosomy 21 linked to mental retardation. However, as the cognitive profile of Dyrk1A+/− mutants has not been examined so far, there is no information available regarding any possible alterations in the cognitive-related processes caused by *Dyrk1A* dosage reduction. The present report investigates the intrinsic ability of Dyrk1A+/− mice to form spatial memories, a function that is disturbed in patients with mental retardation and relies on particular weakness in hippocampal functions. We have selected the Morris water maze task to address the possible abnormalities in spatial memory in Dyrk1A+/− mice to identify the roles of *Dyrk1A*. However, since previous studies [29] suggested that specific mental retardation models might be more responsive to potential stressors and more prone to swim-induced hypothermia, we have also tested our mice in a swimming test designed to determine the influence of the levels of aversiveness associated with the test. Finally we have used other hippocampally-dependent task, such as novel object recognition that has been shown to present profound alterations in Ts65Dn, but not in Ts1Cje Down syndrome mouse models [30], [31]. We have used a simple protocol involving a pair of different objects during the familiarization phase separated from the testing phase by 24 hours, a time frame typically used to evaluate rodent long-term memory. Our experiments indicate that haploinsufficiency of *Dyrk1A* might contribute to the impairment of cognitive functions and adaptative behavior of human monosomy 21.

## Results

### Morris Water Maze

**Figure 1:**
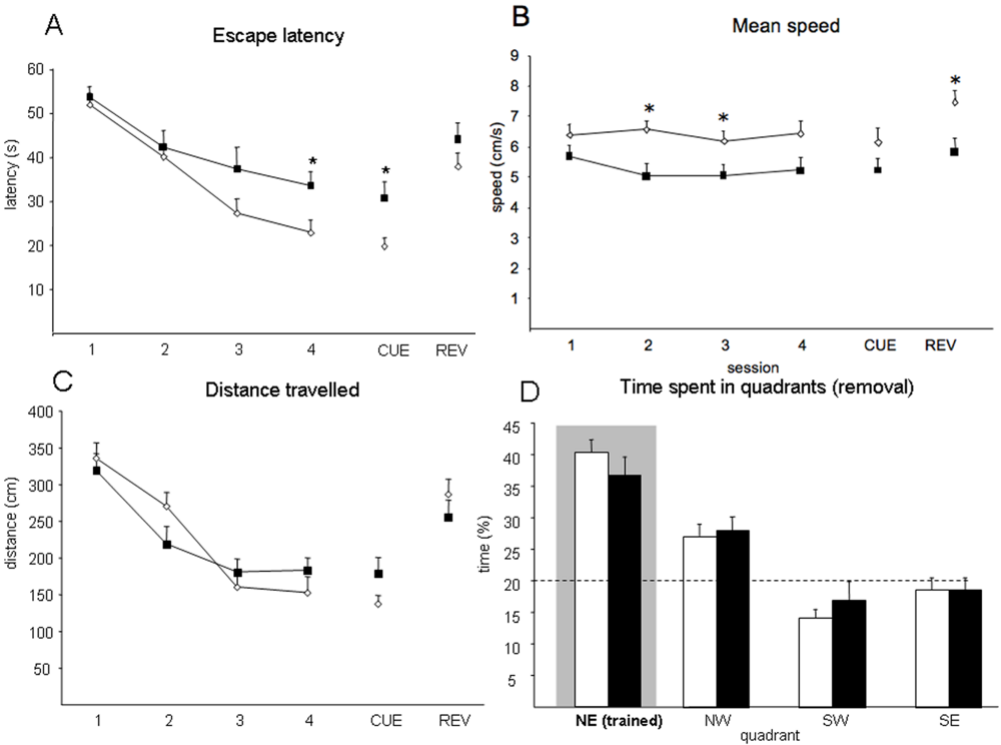
Morris water maze test. Morris water maze performance in Dyrk1A+/− and wild type animals during the learning sessions expressed as (A) latency (s) to find the platform along the acquisition phase, cue and reversal sessions; (B) mean swimming speed; (C) total distance traveled and (D) time spent in the target quadrant during the removal session; discontinuous lines represent the chance level in this session. White bars and circles represent wild types and black bars and circles represent Dyrk1A +/−. Data are represented as mean±SEM; * P<0,05, Student's *t* test. Abbreviations: REV, reversal session; NE, northeast; NW, northwest; SW, southwest; SE, southeast.

In the MWM, both Dyrk1A+/− and wild type mice showed a significant reduction of the escape latency along the four sessions of the acquisition phase (MANOVA, “session”: F_(3–32)_ = 24.2, P<0.0001). However, whereas the ability of wild types to reach the hidden platform improved along the acquisition trials (Fig. 1A), Dyrk1A+/− mice did show an improvement in finding the hidden platform only during the first sessions, but their escape latency differed significantly from the wild types in the last sessions (third session P = 0.058, fourth session P = 0.02), and they thus did not reach the same execution levels. As a consequence, the slope of the escape latency curves for Dyrk1A+/− and wild type mice significantly differed (MANOVA F_(3, 29)_ = 24.22, P<0.05). However, the distance traveled was decreased along acquisition sessions in Dyrk1A+/− mice similar to wild types (Fig. 1C). Swim speed and the use non-spatial search strategies are very important in determining the total swim time. In fact, the average swimming speed of Dyrk1A+/− mice was significantly lower with respect to wild types (MANOVA F_(3, 33)_ = 24.22, P<0.01), thus suggesting that the increased escape latency was due at least in part, to a reduced swimming speed (Fig. 1B). However, it should be noted that swimming speed did not change along acquisition sessions in either group of mice (MANOVA, “session”: F_(3–32)_ = 1.04, P = 0.39) and though significant in some sessions, the differences were not very marked (wild type: 12,15 cm.s^−1^ *vs.* Dyrk1A+/−: 13.14 cm.s^−1^, MANOVA, “session”: F_(1–16)_ = 2,182, P = 0.159). Thus we analyzed both non-searching and non-spatial strategies in our mice (see below).

**Figure 2:**
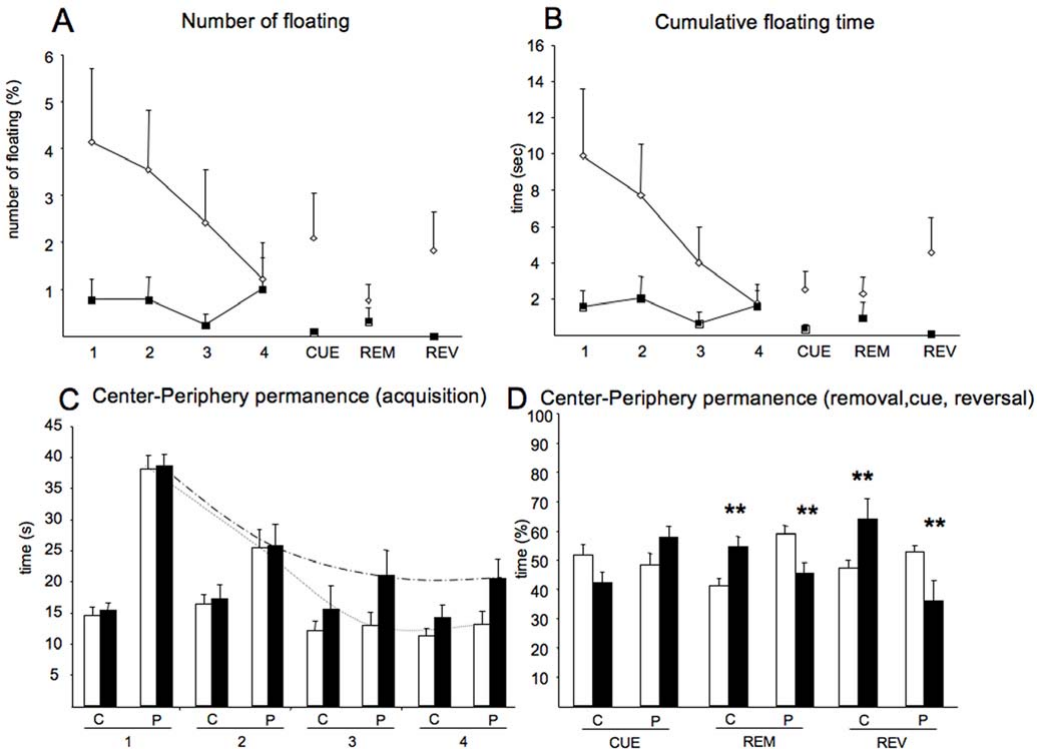
Non-searching strategies in the Morris water maze test. Floating behavior (A and B) was operationally defined as any period equal to or longer than 5 s, during which the average mouse swimming speed stayed below 3 cm.s^−1^. (A) Number of floating episodes; (B) Cumulative floating time (s); (C) Time in center-periphery during acquisition sessions. Dyrk1A+/− (black bars) mice spent more time in peripheral zone than control mice (white bars) during acquisition sessions. Dotted curves show the decrease of time in the peripheral zone between the first and fourth acquisition session. (D) Time (%) in center-periphery calculated for the cued, removal and reversal sessions. Data are shown as mean±SEM. ** P<0,005, Student's *t* test. Abbreviations: REM, removal session; REV, reversal session; C, center; P, periphery.

In the cued session, where the goal was to find a visible platform (black stripped flag), the performance of Dyrk1A+/− mice and wild type littermates did again differ, being the escape latency of Dyrk1A+/− mice significantly longer than that of wild types (*t* = 2.88, P = 0.007, Student's t test, Fig. 1A). To better understand this phenotype we performed a cued version of the water maze (see below). To test whether the mice had indeed learned the spatial location of the hidden platform, and were able to remember this information, the probe trial was conducted after the cued session (4 days after training). During the probe trial (Fig. 1D), the latency to cross the annulus of the hidden escape platform was longer in Dyrk1A+/− mice compared to wild types (wild type: 24.47±3.06 s vs. Dyrk1A+/−: 32.13±4.30 s), although the difference did not reach statistic significance (*t* = −1.08, P = 0.08 Student's *t* test), and the number of crosses was similar between genotypes (wild type, 2.77±0.32; Dyrk1A+/−, 2.01±0.30; *t* = 1.56, P = 0.12, Student's *t* test). Moreover, no differences between genotypes were detected in the preference for the trained quadrant, neither in percentage of time (*t* = 1.04, P = 0.31, Student's *t* test, Fig. 1D) nor in the distance traveled (*t* = 0.66, P = 0.52, Student's *t* test). These observations support the conclusion that Dyrk1A+/− mice indeed remember the location of the platform. One interesting feature during this session is that the speed was significantly increased in both genotypes in comparison to previous sessions (MANOVA, “sessions”: F_(4–32)_ = 3.97, P = 0.01), although again, the speed of Dyrk1A+/− mice was significantly lower than that of wild types (*t* = −5.25, P<0.0001, Student's *t* test). During this session, the distance traveled in the center and periphery of the tank differed significantly between wild types (center: 40.94±2.59; periphery: 59.05±2.59) and mutants (center: 54.52±3.59; periphery: 45.47±3.59) (center: *t* = −3.079, P = 0.004; periphery: *t* = −3.079, P = 0.004, Student's *t* test, Fig 2D).

In the reversal test, that evaluates the cognitive flexibility of the mice, through their ability to learn a new position of the platform, no significant differences were observed between genotypes, neither in the escape latencies (*t* = −1.3, P = 0.19, Student's *t* test, Fig. 1A), nor in the percentage of distance or time swum in each quadrant (*t* = 0.27, P = 0.79, Student's *t* test for the differences per genotype in the distance traveled across the previously trained quadrant). Again, in the reversal session, swimming speed of heterozygous mice was significantly lower (*t* = 2.89, P = 0.008, Student's *t* test, Fig. 1B).

The above results indicate that, although Dyrk1A+/− mice show a hypoactive behavior in the MWM task, they are able to learn the position of the hidden platform at the end of the acquisition task and remember its position, as revealed in the probe trial. However, they show an impaired learning efficiency compared to wild types that does not seem to be dependent on their reduced swimming speed.

### Floating behavior

To gain further insight into the reduced speed Dyrk1A+/− mutants showed in the MWM, we studied the floating behavior of these mice and that of their wild type counterparts. Floating behavior, which is characterized by periods of immobility in the swimming tank, is a phenomenon associated with the performance of the MWM by rodents, particularly mice. Even relatively short intervals of floating behavior can cause significant changes in the mean raw average speed of a trial. We quantified the number of episodes (Fig. 2A) and the average floating time (Fig. 2B) during each trial across genotypes and experimental conditions used in the present MWM experiment. Floating was operationally defined here as any period equal to or longer than 5 s, during which the average mouse swimming speed stayed below 3 cm/s^−1^. Although the analysis of the number of floating episodes (Fig. 2A) or the cumulative floating time (Fig. 2B) did not reveal a significant effect of genotype on floating behavior for the acquisition, visible, probe and reverse platform tasks (*t* = 0.130, P = 0.898, Student's *t* test), a clear difference in the floating pattern along sessions was observed between Dyrk1A+/− and wild type mice. Whereas Dyrk1A+/− mice did not show significant amounts of floating in any of the testing periods, wild types showed a significantly higher percentage and time of floating behaviors that was decreased along acquisition sessions, but increased again in the visible platform and reversal learning sessions (Fig. 2A and 2B). These results suggest that an increased floating behavior cannot account for the reduced swimming speed of Dyrk1A+/− mice in the MWM. One possible explanation for the increased floating behavior in wild type mice could be that rather than being a stress response, the floating behavior may reflect a spatial orientation phase of the animals. To test this hypothesis we determined the occurrence of orientation movements (e.g., turns in the swim path) during such floating period. Only wild type mice did orientation movements during the floating periods (1,5±.01 orientations in wild type).

### Thigmotaxis

To discard other possible non-spatial learning strategies, such as thigmotaxis (swimming along the walls of the pool), we analyzed the time and distances traveled by the mice in the periphery and the center of the tank (Fig. 2C and 2D). This analysis did not reveal differences between genotypes in the first acquisition sessions. However, wild type mice showed a significant decrease in the amount of time spent in the periphery in the last sessions of the acquisition phase, which was not observed in Dyrk1A+/− mutants. In the cued session, Dyrk1A+/− mice spent more time in the periphery than wild types, but the number and the time of floating episodes were reduced. Thus, the worse performance of mutants in the last sessions might be due to an altered searching strategy.

### Learning strategies in the MWM

**Figure 3:**
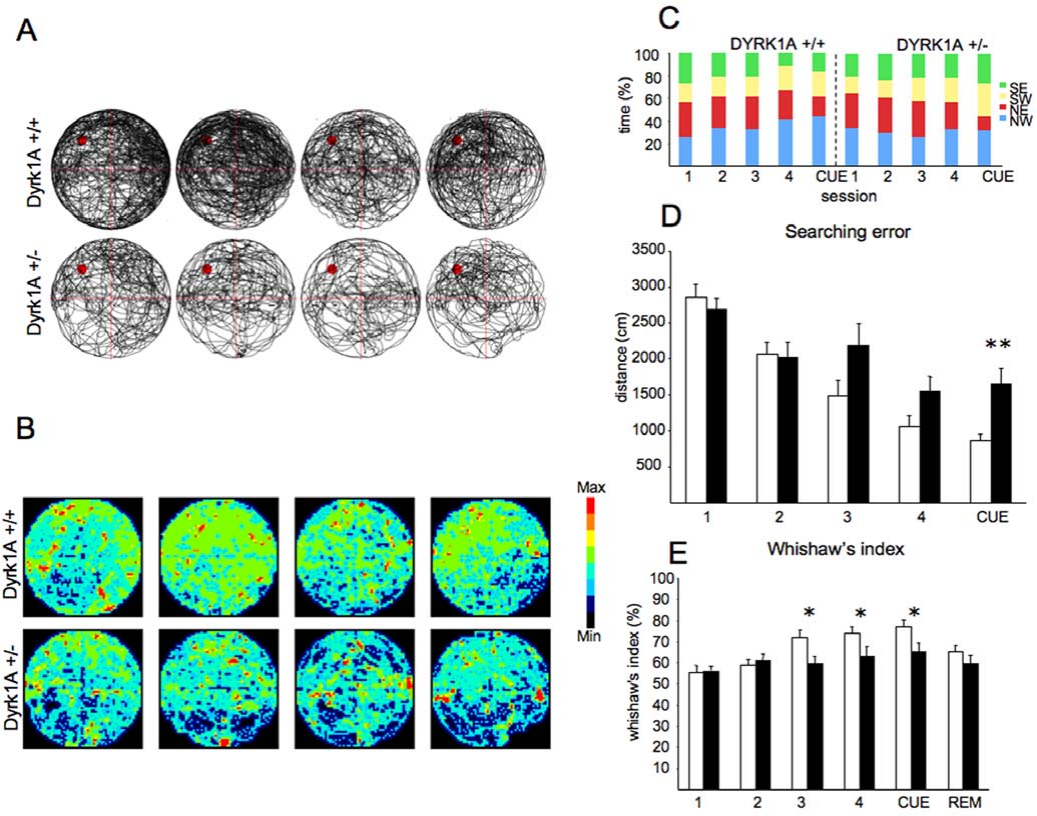
Learning strategies in the Morris water maze test. (A) and (B) represent the spatial preference of Dyrk1A+/− and Dyrk1A+/+ mice along four acquisition sessions. (A) Representative swim paths of a wild type and a Dyrk1A+/− mouse illustrating that Dyrk1A+/− mouse swam more irregularly than the control mouse. (B) Color-coded histograms representing occupancy of wild type (upper panel) and mutant (lower panel) mice during the acquisition sessions of the hidden platform version of Morris Water Maze task. The wild type mice focused their search in the trained location (where the platform used to be during training) whereas the mutant mice visited the whole maze area equivalently. Color scale is given on the right of the histograms. (C) Cumulative permanence in quadrants. Percentage of time in quadrants of mice spends in four acquisition sessions and cue session. Each quadrant is represented by a different color. (D) Cumulative search error. Dyrk1A+/− mice (black bars) and control littermates (white bars) summed one-second averages corrected for the particular start location and platform location by subtracting the proximity score that would be produced by perfect performance on that trial. (E) Whishaw's index. Dyrk1A+/− mice revealed decreased percentage of time spent in correct corridor. Data are shown as mean±SEM (wild types, n = 12; Dyrk1A+/−, n = 11; * P<0,05; **, P<0,005, Student's *t* test). Abbreviation: REM, removal session.

Since the poorer execution of heterozygous mice during the acquisition phase was not directly correlated with the reduced speed (that was constant along sessions) nor with an increased floating behavior, but the searching trajectory of the mice was significantly different, we sought to analyze in detail the learning strategies used by Dyrk1A+/− mice. To this end, we used a customized analysis program, *jTracks* (see Methods section). Fig. 3 shows the scatter plots of the swimming patterns observed in the MWM, showing the integrated tracks (Fig. 3A) and the color-coded frequencies of stage (Fig. 3B), being the most frequently visited areas in red and orange. Plotted along the four acquisition sessions, the swimming patterns show that wild type mice develop a clear spatial preference, whereas heterozygotes distribute their activity similarly across all quadrants, indicating reduced spatial learning. Moreover, a careful analysis of the time of permanence in each quadrant along sessions (Fig. 3C), revealed that Dyrk1A+/− mice did not show the increase in the percentage of time spent in the platform quadrant (NW in blue), that characterizes spatial learning, and that was present in wild types. Dyrk1A+/− showed a similar percentage of time in the trained quadrant along all acquisition sessions (Fig. 3C). Moreover, the calculated searching error for Dyrk1A+/− mice was superior in heterozygous mice in the last acquisition sessions and in the cue session (*t* = −2.747, P = 0.009, Student's *t* test; Fig. 3D). Finally, the Wishaw's index, used to measure the efficiency of the swim paths to reach the goal location (Fig. 3E) revealed a worse spatial learning strategy, as shown by the path traveled within a straight corridor connecting the start and the goal in Dyrk1A+/− mice specially during the last acquisitions sessions (acquisition 3: *t* = 2.17, P = 0.03, Student's *t* test; acquisition 4: *t* = 2.00, P = 0.05, Student's *t* test; Fig. 3E) and in the cued session (*t* = 2.44, P = 0.019, Student's *t* test).

### Swimming test

**Figure 4:**
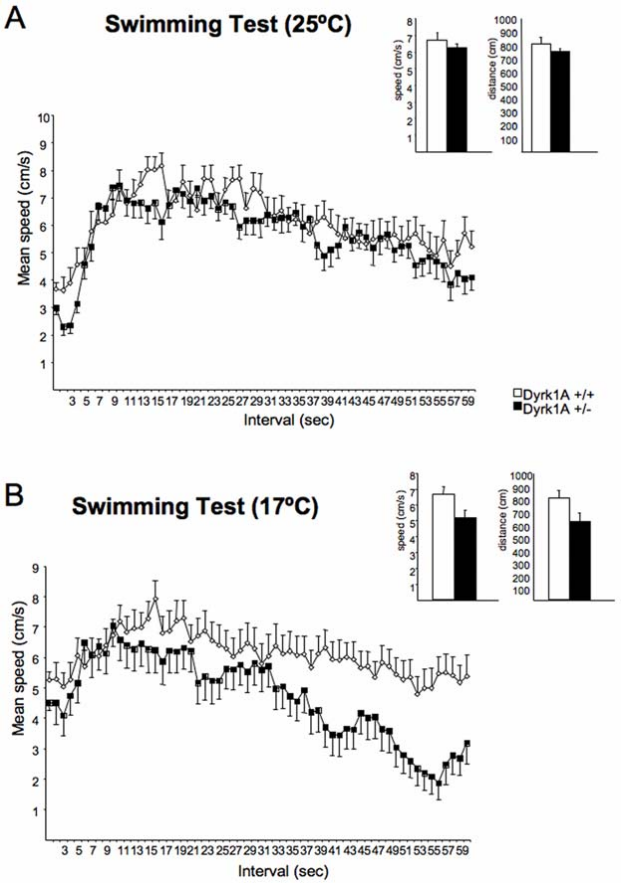
Swimming Test. (A) Swimming test with water temperature at 25°C reveals that the swimming speed between Dyrk1A+/+ and Dyrk1A+/− mice is similar during a 60 s trial period. Inset: Total mean speed and distance traveled by Dyrk1A+/− and wild type mice. (B) Swimming test with water temperature at 17°C reveals a significant reduction of the swimming speed in the mutants compared to wild types, allowing detecting a hypoactive behavior under stressful environmental conditions. Inset: total mean speed and distance traveled by mutant animals mice were less than wild type mice. Open circles (Dyrk1A+/+) and black circles (Dyrk1A+/−) represent means±SEM.

As reported above, during the MWM trials, a significantly reduced average swimming speed was detected in Dyrk1A+/− mice compared to wild types. The swimming speed of mice in the water maze can vary dramatically depending on the environmental conditions, especially the water temperature. However, comparing the swimming speed of a mutant strain of mice with the raw average swimming speeds of control mice during an entire trial does not allow distinguishing between an intrinsic inability to sustain typical levels of swimming speed because of primary motor dysfunction or behavioral and/or neurosensory artifacts. The swimming test, allows to revisit and to expand the specific analysis of motor and stress-related behaviors. In this experiment we have decreased the sampling interval to 1s and analyzed second-to-second variations in swimming speed of Dyrk1A+/− mice in order to improve temporal resolution and potentially capture finer details of the speed variability. We analyzed swimming speed during 60 s in the water tank at 25°C and the following day at 17°C, a temperature that is more stressful for the animal. We decided to use these conditions, since the goal was to analyze the contribution of “despair-like” behaviors to the reduced performance in the navigation task. Fig. 4A displays the raw average swimming speeds during the whole trial as a function of the genotype of animals under non-aversive circumstances (25°C). No genotype-dependent differences in raw average swimming speeds (Fig. 4A inset) were observed and both Dyrk1A+/− and their controls showed a reduction in swimming speed along the 60 s of the trial (MANOVA, “time interval”: F_(1–59) = _13.741, P<0.0001; Fig. 4A). However, at 17°C significant differences were detected in Dyrk1A+/− mice as compared to wild types along the experiment (F_(1–59) = _11.05, P<0.0001; Fig. 4B) and in mean speed (Fig. 4B, inset). As assessed by two-way repeated measures ANOVA (with water temperature and genotype as the main factors), the reduction in water temperature in the swimming tank from 25°C to 17°C produced significant genotype-dependent effect on the swimming speed, which showed a significant interaction between water temperature and genotype. Consequently, when analyzing the “time interval×genotype” of both groups although no significant differences were observed at 25°C (MANOVA, “time interval×genotype”: F_(1–59) = _1.065, P = 0.345), speed was significantly reduced in Dyrk1A+/− mice at 17°C (MANOVA, F_(1–59) = _2.36, P<0.0001). Also, when comparing Fig. 4A (water temperature = 25°C) and Fig. 4B (water temperature = 17°C), the differences between curves of wild type and Dyrk1A+/− mice were generally larger at 17°C than at 25°C. These results show that in non-aversive conditions the swimming speed of Dyrk1A+/− mice is similar to that of wild types. However, under environmental factors that cause stress, mutant mice display a hypoactive behavior.

### Cued version of the Morris Water Maze

The longer latency to reach the platform in the cued session of the MWM, could reflect procedural learning alterations or the presence of other contributing factors to the worse performance in the spatial learning task. Thus, animals were trained in an entirely cued version of the MWM. In this experiment both genotypes learned to reach the cued-platform (Fig. S1) although the escape latency was significantly shorter in wild type than in Dyrk1A+/− mice during the training session (*t* = −2.662, P = 0.022, Student's *t* test; Fig. S1A) and the first test session (*t* = −1.937, P = 0.079, Student's *t* test), but no differences were observed in the distance traveled (Fig. S1B). The retest session 24 hours later, showed similar latencies in both genotypes (*t* = 0.908, P = 0.119, Student's *t* test). This effect might be dependent on a reduced swimming speed during the training and in the 1 hour test session both in center and peripheral zones in Dyrk1A+/− mice (periphery: *t* = 4.249, P = 0.001, center: *t* = 2.607, P = 0.024; Student's *t* test; Fig. S1C), since the analysis of total distance did not show differences along sessions (Fig. S1B).

### Novel object recognition

**Figure 5:**
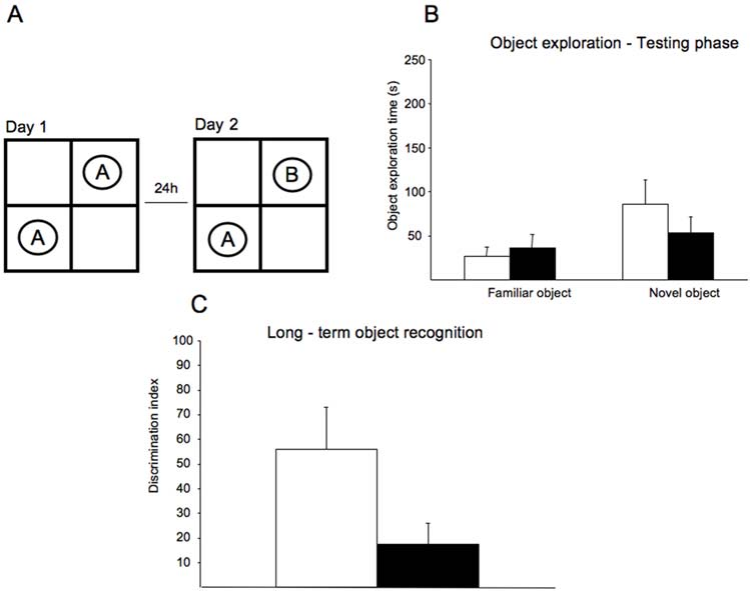
Object recognition test. (A) Schematic representation of the object recognition test. Mice are allowed to explore an identical pair of objects, and after 24 hours, they are presented with the familiar object and a new object. (B) Dyrk1A+/− exhibited significantly impaired novel object recognition as shown by the similar amount of time spent in exploring the two objects (familiar and new). (C) Dyrk1A+/− showed no net preference between novel and familiar objects as shown by the reduced discrimination index. White bars (Dyrk1A+/+) and black bars (Dyrk1A+/−) represent means±SEM.

The hippocampus is important for spatial memory, but its integrity is also necessary for recognition memory [32]. Besides, Fernandez et al (2007) [30] described clear disturbances in this hippocampal function in a DS mouse model, the Ts65Dn partial trisomic mouse. We tested Dyrk1A+/− mice in a novel object recognition task that relies on the mouse's natural exploratory behavior. Fig. 5A shows the schematic representation of the protocol, in which mice are habituated to the open-field apparatus, on day 1 they were allowed to explore two identical objects, and after 24 hours, they were presented with the familiar and a new object. Dyrk1A+/− mice exhibit significantly impaired novel object recognition performance in the simple task relative to wild type mice (85,33±27,92 in wild type vs. 52,8±18,96 seconds exploring the novel object in Dyrk1A+/−; *t* = 0.968, P = 0.158, Student's *t* test; Fig. 5B). Consistent with the lack of net preference between novel and familiar objects, the discrimination index (DI = (Novel Object Exploration Time/Total Exploration Time)–(Familiar Object Exploration Time/Total Exploration Time)×100) in mutant mice was reduced with respect to wild types (*t* = 1.774, P = 0.054, Student's *t* test; Fig. 5C).

## Discussion

The present study is the first to address the possible learning phenotypes in mice haploinsufficient for *Dyrk1A* and the impact of various test conditions on their performance. *DYRK1A* is a candidate gene for Down syndrome localized on the human chromosome 21q22.2 region [6]. At this chromosomal region Chettouh et al. [33] mapped the putative loci for intrauterine and postnatal growth retardation, microcephaly, low-set ears, cleft palate and mental retardation in monosomy 21. Moreover, Møller et al [18] have reported two unrelated cases in which a *de novo* balanced translocation that truncates the *DYRK1A* gene gives rise to microcephaly, intrauterine growth retardation, feeding problems, developmental delay, and febrile seizures/epilepsy. Of interest to the monosomy phenotype, Dyrk1A heterozygote mice show decreased neonatal viability, pre-weaning developmental delay and specific motor and behavioral deficits in adults [19]. We demonstrate here that reduced dosage of *Dyrk1A* impedes the use of efficient learning strategies, gives rise to reduced recognition memory, and produces certain genotype-dependent features, related to an increased susceptibility to swimming-temperature.

In the present experiments, the MWM [34] was performed according to the “hidden–visible” platform sequence. This task that addresses abnormalities in visuo-spatial memory and has proven useful to detect hippocampal-dependent cognitive deficits being a suitable tool to identify genes with critical roles in mental retardation [23], [27]–[29]. The hippocampus has been implicated in both spatial and contextual learning and in emotional behavior in rodents [35], and is a main brain structure affected in mental retardation [1], [22], [36]–[38]. In the learning phase of the MWM, Dyrk1A+/− mice showed a reduced efficiency in the execution of a standard spatial learning task, revealed by their inability to reach similar asymptotic execution levels, to those attained by wild type littermates. It should be noted though, that reduced dosage of *Dyrk1A* did not completely prevent spatial learning, as indicated by the initial trials of the place acquisition task. One possibility in the present experiments was that the impairment observed was in fact contributed by other factors, or the use of non-spatial learning strategies, as has been also demonstrated in other mouse models [29]. In this regard, previous works in our laboratory showed that the Dyrk1A+/− phenotype is characterized by a marked hypoactivity [20]. This was also the case in the water maze experiment, since Dyrk1A+/− mice showed a constant reduction in swimming speed, that could contribute to the differences in escape latency, since distance was less affected. However, even though the reduced speed was constant along all sessions, escape latencies only showed significant differences in the last two acquisition sessions. This could be attributed to the fact that wild type mice reduced their tigmotactic behavior along sessions, whereas Dyrk1A+/− mice persisted in the use of this non-spatial strategy. Moreover, both groups displayed very few behaviors indicative of sensorimotor impairments during place task performance; i.e., although escape latencies were increased in Dyrk1A+/− mice, there were no differences in failing to climb onto the platform, or jumping off the platform.

To investigate further if impaired learning strategies could account for the observed deficits we performed a careful analysis of the swimming trajectories. This analysis showed that spatial preference for the platform quadrant was only developed by wild type mice, whereas heterozygous mice distributed their activity similarly across all quadrants, indicating reduced spatial learning. As a consequence, permanence time in the target quadrant did not increase along sessions in Dyrk1A+/− mice (Fig. 3C). Moreover, the calculated searching error and the Wishaw's index corresponding to the percentage of path traveled within a straight corridor connecting the start and the goal, was increased in Dyrk1A+/− mice thus most probably contributing to their worse performance. These results indicate that *Dyrk1A* dose reduction may contribute to impairment in the development of adequate spatial learning strategies, although it does not completely abolish the learning capacities of these mice, thus suggesting that the correct dosage of *Dyrk1A* is necessary for the adequate performance of the spatial learning task. It should be borne in mind, however, that transgenic mice overexpressing *Dyrk1A* show a more severe learning phenotype [21], [23]. Since our previous work also showed more severe alterations in the Ts65Dn model, that bears *Dyrk1A* in trisomy [27], [28], the present results support the notion that *Dyrk1A* is involved in spatial learning in a dosage-sensitive manner. Regarding the spatial memory component of the task, that is explored during the probe trial, even though Dyrk1A+/− mice demonstrated certain knowledge of the correct quadrant, thus discarding other non-cognitive problems, such as vision problems, they made significantly fewer target crossings over the exact location where the platform had originally been located than wild types.

In view of the relatively slight effects on the MWM paradigm, and since alterations in other hippocampal-dependent tasks have been described in DS models [30], [31] we performed a novel object recognition test. Previous work showed that Ts65Dn and Ts1CjE mice react normally to object novelty over short intervals of a few minutes, but cannot detect object novelty over 24 hours (a typical time frame used to evaluate rodent long-term memory) [30], [31]. Interestingly, people with DS often present learning problems thought to result from failures to “stabilize” or consolidate information after initial acquisition [36]. In our experiments Dyrk1a+/− mice showed a clear impairment of novel object recognition performance in the simple task relative to wild type mice. Taken together the results obtained in the MWM and object recognition tests suggest that the correct dosage of *Dyrk1A* is necessary to efficiently perform hippocampal-dependent tasks. At a physiological level, the involvement of *Dyrk1A* in hippocampal function has been demonstrated using transgenic mouse models [21], [39], [40]. Comparing four different mouse transgenic lines overexpressing different regions of human chromosome 21, only mice from line 152F7 revealed behavioral and morphological abnormalities, which were attributed to *Dyrk1A* [40]. In addition to learning deficits [39], [40], these mice displayed an increase in the size of the cortical and hippocampal cell bodies with a subsequent alteration in the morphology of the nuclei [40]. Interestingly, 152F7 mice showed changes in the levels of phosphorylated CREB, with a significant increase at early postnatal stages compared to control animals [40]. These data provide *in vivo* evidence associating *Dyrk1A* expression levels with CREB phosphorylation, hippocampal morphological aberrations and learning deficits. They also underline the complexity of these relationships, as increase in the levels of *Dyrk1A* during postnatal development leads to increased and later reduced levels of phosphorylated CREB [40 and see below]. In fact, regarding the possible structural correlates, the brains of heterozygous mice are decreased in size of specific regions [19], and cortical neurons present alterations in their cytoarchitecture [22] that may lead to impairment of information processing in the cerebral cortex. No gross morphological abnormalities have been observed in the hippocampus of Dyrk1A+/− mice, but detailed morphometric analysis needs to be performed to reveal whether defects in this structure, similar to those in the cortex, may associate *Dyrk1A* deficiency with hippocampal dysfunction. In support of this hypothesis, the learning and memory pattern observed in Dyrk1A+/− mice is similar to hippocampally-lesioned animals that also learn at a slower rate than sham-lesioned animals [41], [42], but show partially preserved memory. It has been suggested that the DYRK family shares biochemical similarities to the mitogen activated protein kinases (MAPK) [43] and recently a bioinformatics approach has proposed that human *DYRK1A* may belong to a novel MAPK cascade [44]. Although it is not yet clear in which molecular pathway(s) *Dyrk1A* is involved, several different classes of substrates have been found to be phosphorylated by this kinase [reviewed in 45]. Among them and with relevance to hippocampal function is the cAMP-response-element-binding protein (CREB) [46]. In fact, *Dyrk1A* phosphorylates CREB on serine 133, enhancing CREB mediated transcription during neural differentiation in hippocampal cells [46]. Nevertheless, the brain alterations observed so far in Dyrk1A+/− mice [19], [22] may affect their ability to complete a simple water-escape task requiring swimming and spatial skills, while additional hippocampal related subtle changes might also be implicated in these defects.

One of the possible confounding elements in our experiments is the reduced swimming speed that was a relatively important component in Dyrk1A+/− mice and may suggest that other neural systems, such as different areas of the cerebral cortex (perirhinal, parietal cortex, frontal or cingulated cortex) [47], 48, the subiculum [49], or the medial striatum [50], [51] may also participate in the observed impairment. Reduced swimming speed could be dependent on increased floating behavior, that has been considered a non-searching strategy [29]. However, the analysis of floating bursts revealed that floating was not predominant in heterozygous mice. This reduced floating is a striking observation, in view of the poorer performance of mutant mice in the MWM. Thus, we analyzed the occurrence of orientation movements (e.g., turns in the swim path) during such floating periods. The presence of orientation movements was only detected in wild type animals, suggesting the possibility that the floating behavior detected in our experiments may reflect a spatial orientation phase of the animals.

However, since floating behavior may also reflect a stress-related response, being thus a potential confounding factor in the interpretation of these results, we attempted to modulate the degree of aversiveness associated with the water maze task by using a swimming test in which we could decrease the temperature of the water in the same pool used for the water maze, from 25°C to 17°C. Thus, the experimental design used in the swimming test shared some of the characteristics of the Porsolt swimming test [52], with the exception of the size of the pool. Surprisingly, under the less aversive circumstances (25°C) that were the same used in the spatial learning task (water maze), no genotype-dependent differences in average swimming speeds are observed, contrary to the results attained when the escape platform was present in the pool. Although this result is difficult to interpret, it may be argued that the different performance of Dyrk1A+/− mice may be dependent on an altered reactivity to situations with a stress component. Confirming this hypothesis significant genotype-dependent differences were detected in swimming speed when the temperature was reduced to 17° in the swimming test (Fig. 4). Thus, the present results indicate that genotype-dependent differences in raw swimming speed observed by us and others are most likely the result of behavioral phenomena affecting swimming patterns rather than a direct effect of motor dysfunction affecting swimming in Dyrk1A+/− mice.

In conclusion, the reduced performance levels in the spatial navigational task provide evidence about the role of *Dyrk1A* in spatial learning in mice and support a role for *Dyrk1A* in the hippocampally-mediated interaction between stress and cognitive performance. We thus propose that *Dyrk1A* is a dosage-sensitive gene that is necessary to form spatial learning and memory storage and provide a further functional link between human *DYRK1A* and partially monosomy 21. Taken together, our results suggest that specific phenotypes associated with monosomy arise from the removal of critical, haploinsufficient genes such as *DYRK1A*.

## Materials and Methods

### Animals

Generation of *Dyrk1A* mutant mice has been described previously [19]. Mice heterozygous for the mutation (Dyrk1A*+/−*) were maintained in a C57BL/6J-129Ola (C57-129) mixed genetic background. Experiments were done using Dyrk1A*+/−* mice and wild-type (Dyrk1A*+/+*) littermates obtained by crossing F1:C57-129 wild-type females (Harlan Ibérica, S.L.) with C57-129 Dyrk1A+/− males. Same sex littermates were group-housed (4–6 animals per cage) in standard macrolon cages (40×25×20 cm) under a 12-h light/dark schedule (lights on 0600 to 1800) in controlled environmental conditions of humidity (60%) and temperature (22±2°C) with food and water supplied *ad libitum*. All the behavioral testing was conducted by the same experimenter in an isolated room and at the same time of the day. Behavioral experimenters were blinded as to the genetic status of the animals. The Morris water maze test and the swimming test were performed with separate group of mice to exclude influence between tests. Standardized handling protocols were administered three days before testing to minimize the influence of the experimenter. All animal procedures met the local guidelines (Spanish law 9/2003, and Catalan law 5/1995), European regulations (EU directive n° 86/609, EU decree 2001-486) and Standards for Use of Laboratory Animals n° A5388-01 (NIH). Researchers had a specific qualification for experimentation on live animals.

### Behavioral tests

#### Morris Water Maze test

To test hippocampal-dependent spatial cognition, Dyrk1A+/− mice were trained in the standard Morris water maze (MWM) with a hidden platform [34]. 23 wild type and 13 Dyrk1A+/− mice were tested over 4 days (4 trials/session, 10-min inter-trial intervals). The water maze consisted of a circular pool (diameter, 1.20 m; height, 0.5 m). It was filled with tepid water (24°C) opacified by the addition of powdered milk (0.9 kg). A white escape platform (15 cm diameter, height 24 cm) was located 1 cm below the water surface in a fixed position (NE quadrant, 22 cm away from the wall). In each trial, mice were placed at one of the starting locations in random order [north, south, east, west (N, S, E, W), including permutations of the four starting points per session] and were allowed to swim until they located the platform. Mice failing to find the platform within 60 s were placed on it for 20 s (the same period of time as the successful animals). At the end of every trial the mice were allowed to dry for 15 min in a heated enclosure and were returned to their home cage. The cue session was performed to test the swimming speed and visual ability using the visible platform, elevated 1 cm above the water and its position was clearly indicated by a visible cue (black flag). White curtains with affixed black patterns to provide an arrangement of spatial cues surrounded the maze. It was performed 24 hours after the fourth training sessions and 5 days after completion of the hidden platform training protocol. To test whether the mice remembered the location of the platform, probe trials were performed. In the probe session the platform was removed and mice were allowed to swim for 60 s. The time spent in the trained and non-trained quadrants as well as the number of platform annulus crossings during 60 s were recorded. On the next day (5 days after the last acquisition session), mice performed the reversal learning session. In this test, the platform position was changed to the opposite quadrant (SW).

All the trials were recorded and traced with an image tracking system (SMART, Panlab, Spain) connected to a video camera placed above the pool. Escape latencies, length of the swimming paths and swimming speed for each animal and trial were monitored and computed. Path length was defined as the total distance swum from the start location to the target and latency as the total duration of the trial from when the mouse was placed in the water until it located the escape platform. To better evaluate the spatial distribution of the behavior of the mice, the paths traveled in peripheral (15 cm wide) and central rings were measured in each trial. Several measures were used to assess accuracy of spatial learning in the water maze. The primary measures were cumulative search-error on training trials and a learning index (Gallagher's proximity index) computed from the trials given over the course of training. These measures rely on a computation of distance from the platform during the trial (Fernandez et al., unpublished). Briefly, to quantify proximity of the animal to the target of a water maze over the course of the search (Gallagher's proximity index [53]) the distance from the platform is sampled 5 times per second during the trials; these distances are averaged in 5 s bins. Swim trajectory errors were measured as the inability of a mouse to swim in a relatively direct path from the start position to the location of the hidden platform [54], [55]. A correct score (assigned a value of 100) was obtained when the subject swam directly to the platform while remaining within a 20 cm wide corridor, extending from the start location to the platform. Swimming outside the 18-cm corridor resulted in an incorrect score (given a value of 0). Gallagher's cumulative distance and Gallagher's average proximity from the goal were calculated using the SMART® video-tracking software and a custom-designed analysis program, jTracks (Fernandez D. et al., unpublished). The aim of this software is to expand the SMART© analysis by providing Gallagher proximity index, cumulative searching errors, distance traveled, escape latency, Whishaw's index, permanence time in quadrants or in center-periphery, average speed per areas and floating and to provide graphic representational tools.

### Cued version of the MWM

To carefully analyze the possible factors involved in the worse performance of Dyrk1A+/− in the MWM, a separate group of Dyrk1A+/− (n = 6) and wild type mice (n = 7) were tested in an entirely cued version. The water maze apparatus and experimental conditions are the same than in the spatial MWM protocol. One training session (four trials) was performed in which the platform was located in the center of the apparatus, protruding above the surface of the water. At the beginning of each trial, mice were placed in the maze facing the wall at one of the different starting positions [north, south, east, west (N, S, E, W), including permutations of the four starting points per session]. They were allowed to swim freely or until they reached the platform. Mice failing to find the platform within a fixed period of 60 seconds were gently guided by hand to the platform and a maximum escape latency of 60 seconds was recorded. After the animals had climbed onto the platform, they were allowed to remain on it for additional 20 seconds. Mice were then submitted to two consecutive test sessions of four trials with an inter-session resting period of 15–20 minutes during which they were returned to their home cage. On test sessions, the escape platform was located in a fixed position (NE quadrant, 22 cm away from the wall), and had a 10 cm height visible cue (black flag) to indicate its location. The first test session was performed 1 hour after the training session 24 hours later the second test session was performed. Escape latencies, length of the swimming paths and swimming speed for each animal and trial were monitored and computed.

### Swimming Test

The level of aversiveness associated with the navigation test is one important experimental parameter that may influence the performance of mice in the cognitive tests . The swimming test allows the detection of changes in the motor activity in an environment of variable aversiveness due to extrinsic factors, as the temperature of the water. The apparatus was the same pool used in the MWM experiments. To maintain the same experimental conditions in the navigation task as in the MWM, milk was diluted to obtain a white opaque color and to avoid the distraction of the animal. The task consisted of two sessions that were performed along two consecutive days. During the first session (day 1), each mouse was allowed to swim during 60 s in the tank at temperature of 25°C. In the second session (day 2), the time of swimming was again 60 s, but the constant temperature of the water was diminished to 17°C. We recorded the average swimming speed of each group sampled once per s during the trials.

### Object Recognition Task

The novel object recognition task is based on the innate tendency of rodents to differentially explore novel objects over familiar ones. Mice were placed into an open-field (OF) apparatus consisted of a rectangular area (70 cm wide×90 cm long×60 high) made of metacrylate. In the training trial (familiarization phase) the animals were presented with a pair of identical objects until they had explored the objects during 20 seconds, in a maximum period of 15 minutes. The exploration of the objects is considered as any investigative behavior (head orientation or sniffing occurring) or deliberate contact that occurred with each object in a distance < or = 2 cm or when touching with the nose. In the testing trial (the test phase), performed 24 hours later, one of the familiar objects was changed for another new, and the animals were left in the OF during 15 min. The exploration time for the familiar (TF) or the new object (TN) during the test phase was recorded. Memory was operationally defined by the discrimination index for the novel object (DI) as the proportion of time animals spent investigating the novel object minus the proportion spent investigating the familiar one in the testing period [Discrimination Index, DI = (Novel Object Exploration Time/Total Exploration Time)–(Familiar Object Exploration Time/Total Exploration Time)×100]. We also register activity parameters such speed, distance and the time spent in the center and the periphery of the apparatus. To control for odor cues, the OF arena and the objects were thoroughly cleaned with 10% odorless soap, dried, and ventilated for a few minutes between mice.

### Data analysis

Performance on the Swimming Test and on the MWM was compared using MANOVA. Simple comparisons between Dyrk1A+/− mice and wild types in various tasks were performed using the two-tailed unpaired Student t-test with Mann-Whitney's correction to account for the different variances in the populations being studied. Data were expressed as mean and ±SEM. Mean and cumulative Gallagher distances, escape latency, traveled distance and permanence in quadrants were calculated using the jTracks software. In all tests, a difference was considered to be significant if the obtained probability value was P<0.05. Thus as, a value 0,08<P>0,05 was considered like a significant tendency. The statistical analysis was performed using the SPSS 12.0 software.

## Supporting Information

Figure S1 Cued version of the Morris water maze test. (A) Escape latencies in the training, and 1 hours and 24 hours test sessions in Dyrk1A+/− and wild type mice. B) Total distance traveled during sessions in both genotypes. C) Mean distance in center and periphery of the pool. The white bars and circles (Dyrk1A+/+) and black bars and circles (Dyrk1A+/−) represent means±SEM; * P<0,05; **, P<0,005; Student's t test. Abbreviations: C, center; P, periphery.
(0.20 MB TIF)
