# Supplementary figures and images for: Impaired Spatial Learning Strategies and Novel Object Recognition in Mice Haploinsufficient for the Dual Specificity Tyrosine-Regulated Kinase-1A (Dyrk1A)
Source: PLoS One. 2008 Jul 2;3(7):e2575. doi: 10.1371/journal.pone.0002575 (PMC2481280; doi:10.1371/journal.pone.0002575)

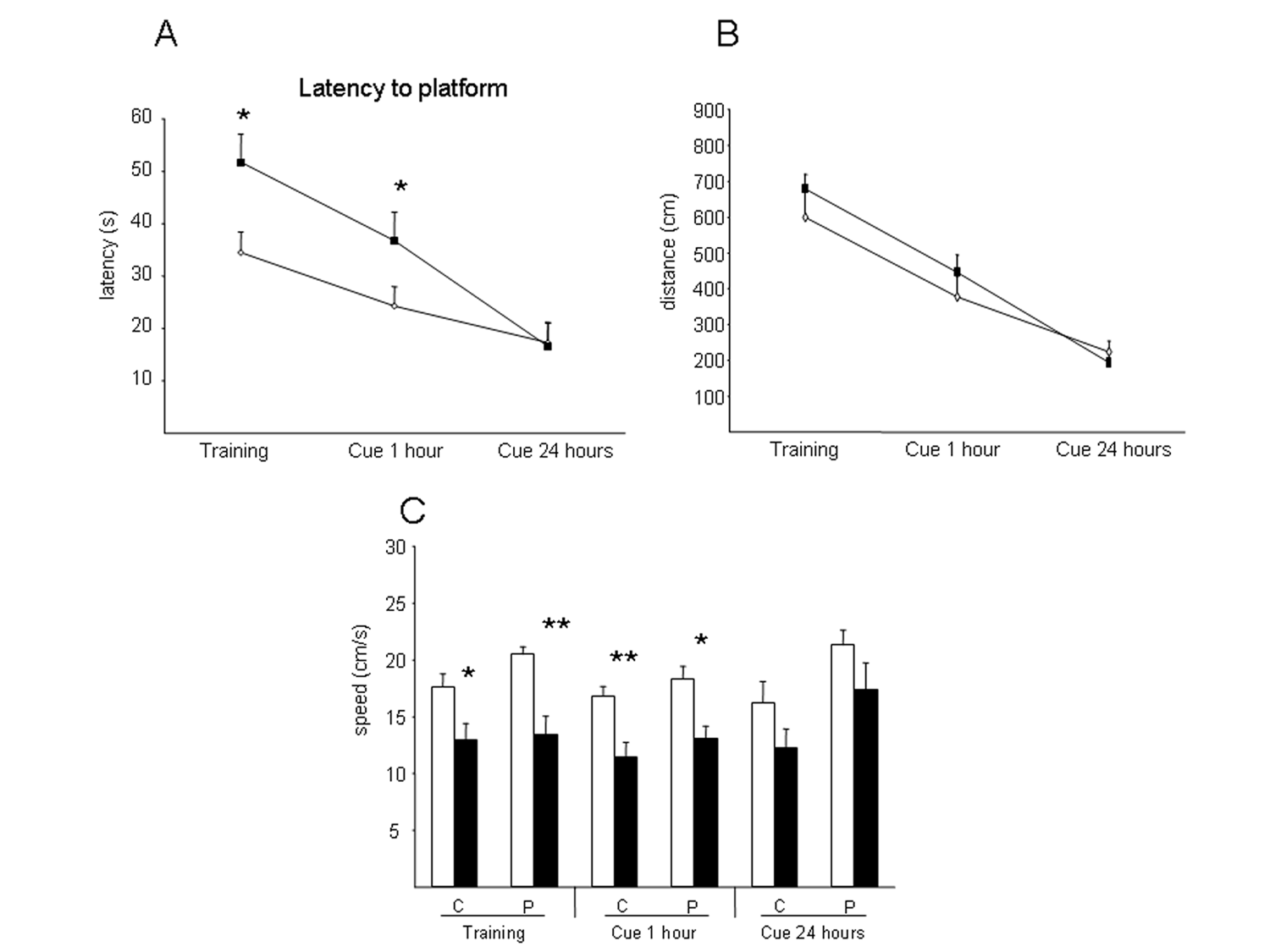

Supplement: Figure S1 — Cued version of the Morris water maze test. (A) Escape latencies in the training, and 1 hours and 24 hours test sessions in Dyrk1A+/− and wild type mice. B) Total distance traveled during sessions in both genotypes. C) Mean distance in center and periphery of the pool. The white bars and circles (Dyrk1A+/+) and black bars and circles (Dyrk1A+/−) represent means±SEM; * P<0,05; **, P<0,005; Student's t test. Abbreviations: C, center; P, periphery. (0.20 MB TIF) [file pone.0002575.s001.tif]
